# Respiratory syncytial virus and other respiratory virus infections in residents of homeless shelters – King County, Washington, 2019–2021

**DOI:** 10.1111/irv.13166

**Published:** 2023-06-19

**Authors:** Denise J. McCulloch, Julia H. Rogers, Yongzhe Wang, Eric J. Chow, Amy C. Link, Caitlin R. Wolf, Timothy M. Uyeki, Melissa A. Rolfes, Emily Mosites, Jaydee Sereewit, Jeffrey S. Duchin, Nancy K. Sugg, Alexander L. Greninger, Michael J. Boeckh, Janet A. Englund, Jay Shendure, James P. Hughes, Lea M. Starita, Pavitra Roychoudhury, Helen Y. Chu

**Affiliations:** ^1^ Department of Medicine, Division of Allergy and Infectious Diseases University of Washington Seattle Washington USA; ^2^ Vaccine and Infectious Disease Division Fred Hutchinson Cancer Center Seattle Washington USA; ^3^ Department of Epidemiology University of Washington Seattle Washington USA; ^4^ Division of Influenza National Center for Immunization and Respiratory Diseases, Centers for Disease Control and Prevention Atlanta Georgia USA; ^5^ Office of the Deputy Director for Infectious Diseases Centers for Disease Control and Prevention Atlanta Georgia USA; ^6^ Department of Laboratory Medicine and Pathology University of Washington Seattle Washington USA; ^7^ Public Health—Seattle & King County Seattle Washington USA; ^8^ Department of Medicine University of Washington Seattle Washington USA; ^9^ Department of Laboratory Medicine and Pathology, Division of Virology University of Washington Seattle Washington USA; ^10^ Seattle Children's Research Institute Seattle Washington USA; ^11^ Department of Genome Sciences University of Washington Seattle Washington USA; ^12^ Brotman Baty Institute for Precision Medicine Seattle Washington USA; ^13^ Allen Discovery Center for Cell Lineage Tracing Seattle Washington USA; ^14^ Howard Hughes Medical Institute Seattle Washington USA; ^15^ Department of Biostatistics University of Washington Seattle Washington USA

**Keywords:** homeless youth, human, ill‐housed persons, influenza, respiratory syncytial virus infections, respiratory syncytial viruses

## Abstract

Respiratory syncytial virus (RSV) causes disproportionate morbidity and mortality in vulnerable populations. We tested residents of homeless shelters in Seattle, Washington for RSV in a repeated cross‐sectional study as part of community surveillance for respiratory viruses. Of 15 364 specimens tested, 35 had RSV detected, compared to 77 with influenza. The most common symptoms for both RSV and influenza were cough and rhinorrhea. Many individuals with RSV (39%) and influenza (58%) reported that their illness significantly impacted their ability to perform their regular activities. RSV and influenza demonstrated similar clinical presentations and burden of illness in vulnerable populations living in congregate settings.

## 1 INTRODUCTION

Respiratory syncytial virus (RSV) can cause severe disease in individuals of all ages, particularly young children and older adults with chronic medical conditions. Adults with asthma, chronic bronchitis, and heart disease experience higher hospitalization rates for RSV infection, and longer hospitalizations.^1^ These same conditions that predispose to more severe RSV disease are more common among people experiencing homelessness (PEH).^2^, ^3^, ^4^ Furthermore, the aging of the homeless population^5^ means that a greater proportion of these individuals are entering a particularly vulnerable demographic.

Among PEH, these medical vulnerabilities are compounded by social vulnerabilities including limited access to medical care and crowded living spaces, which facilitate the spread of respiratory viruses. Studies of SARS‐CoV‐2 outbreaks in homeless shelters demonstrated up to 90% attack rates once the virus was introduced.^6^

Multiple RSV vaccines and preventive monoclonal antibodies are anticipated to be licensed for use shortly, including RSV vaccines targeted toward older, high‐risk individuals and pregnant persons. Data on the epidemiology and clinical presentation of RSV infections among PEH are scarce but are important for decisions around vaccine prioritization in vulnerable populations. The goal of this study was to describe the clinical characteristics and burden of disease of RSV infection among PEH.

## 2 METHODS

### 2.1 Study design and population

We used a repeated cross‐sectional design of active respiratory virus surveillance to enroll residents and staff of homeless shelters in King County, Washington over the course of three RSV seasons from January 2019 to May 2021. Participants were enrolled through two larger studies: the Seattle Flu Study from November 2018 to May 2019,^7^ and a randomized controlled trial of on‐site testing and treatment for influenza in homeless shelters^8^ from November 2019 to May 2021.

Participants were recruited via kiosks at 15 shelters throughout King County, WA, which were staffed 3 to 6 days per week. Through March 31, 2020, shelter residents ≥3 months of age with new or worsening cough, or ≥2 new or worsening symptoms of acute respiratory infection (ARI) in the past 7 days were eligible for inclusion. Eligible ARI symptoms included subjective fever, cough, sore throat, shortness of breath, myalgia, headache, and rhinorrhea. We also offered enrollment regardless of symptoms once monthly through this date. Due to SARS‐CoV‐2 community transmission, participant eligibility expanded to include shelter staff on April 1, 2020. Participants were not followed longitudinally; eligible individuals were allowed multiple encounters. Each encounter included a questionnaire and a nasal swab, and had to occur ≥7 days from a previous encounter, unless they developed new respiratory symptoms <7 days from their prior symptoms. Participants or their proxies (if aged <18) were consented at each encounter.

### 2.2 Data collection

Participants completed symptom screening and enrollment questionnaires electronically on a tablet using Research Electronic Data Capture (REDCap).^9^ Participants then provided a mid‐nasal specimen, obtained using a sterile nylon flocked nasal swab (Copan Diagnostics) collected either by study staff (before 6 March 2020) or self‐collected (after 6 March 2020).

### 2.3 Respiratory viral testing and genomic sequencing

Swabs were placed in tubes containing universal transport medium at ambient temperature, aliquoted, stored at 4°C and tested by a custom arrayed RT‐PCR platform for respiratory viruses as previously described.^10^ SARS‐CoV‐2 testing was added on 25 February 2020.^11^ Sequencing was attempted on all specimens positive for RSV‐A or RSV‐B targets using a metagenomic or capture‐based approach described previously (supplemental methods).^12^, ^13^

### 2.4 Statistical analysis

Statistical analyses were performed using R version 4.1.1. Numerical variables were reported as median (range), and categorical variables were reported as *n* (%). We used *χ* ^2^ tests to compare medical care seeking and limitations on daily activities between those with RSV and influenza infections.

### 2.5 Ethics approval

This study was approved by the University of Washington Institutional Review Board.

## 3 RESULTS

Between January 2019 and May 2021, there were 15,364 encounters. Median age was 41 years (IQR, 25–55) (Table 1); 60% were male and 21.9% were shelter staff. The most common comorbidities wase asthma (12%); 2.5% reported bronchitis and 3% COPD. Participants in 54% of adult encounters reported current tobacco use.

**TABLE 1:** Demographic and clinical characteristics of study encounters.

Participant characteristics	All encounters^a^ (*N* = 15,364)	Encounters with RSV^b^ detected (*N* = 35)	Encounters with influenza detected (*N* = 77)
	*n* (%)	*n* (%)	*n* (%)
Age in years: median (range)	41 (0, 97)	53 (0, 67)	34 (0, 81)
<5	651 (4.0)	4 (11.4)	10 (13.0)
5–11	885 (6.0)	2 (5.7)	7 (9.1)
12–17	506 (3.0)	1 (2.9)	1 (1.30)
18–49	7960 (52.0)	9 (25.7)	37 (48.1)
50–64	4348 (28.0)	15 (42.9)	18 (23.4)
≥65	1011 (7.0)	4 (11.4)	4 (5.2)
Male sex	9214 (60.0)	21 (60.0)	51 (66.2)
Race			
White	6782 (44.1)	16 (45.7)	40 (52.0)
Black	5512 (35.9)	13 (37.1)	30 (39.0)
Asian	649 (4.2)	1 (2.9)	3 (3.9)
American Indian or Alaskan Native	1104 (7.2)	4 (11.4)	2 (2.6)
Native Hawaiian or Pacific Islander	713 (4.6)	2 (5.7)	1 (1.3)
Other	974 (6.3)	4 (11.4)	6 (7.8)
Prefer not to say	1051 (6.8)	1 (2.9)	0 (0)
Hispanic ethnicity	1958 (12.7)	5 (14.3)	8 (10.4)
Comorbidities^c^			
None	10 123 (65.9)	16 (60.0)	43 (55.8)
Asthma	1825 (11.9)	1 (2.9)	12 (15.6)
Bronchitis	382 (2.5)	0(0)	1 (1.3)
Chronic obstructive pulmonary disease	518 (3.3)	0(0)	3 (3.9)
Diabetes mellitus	1252 (8.2)	1 (2.9)	13 (16.9)
Heart condition	503 (3.3)	0(0)	11 (14.3)
Immunocompromised	183 (1.2)	0(0)	1 (1.3)
Liver disease	411 (2.7)	0(0)	1 (1.3)
Missing	900 (5.9)	15 (42.9)	13 (16.9)
Tobacco use	7180 (46.7)	22 (62.9)	43 (55.8)
Self‐reported influenza vaccination (current season)	6770 (44.1)	16 (70.59)	31 (41.33)
Virus type or subtype			
A	N/A	15 (42.9)	34 (44.2)
B	N/A	21 (60.0)	44 (57.1)
Symptomatic	3653 (23.8)	31 (88.6)	66 (85.7)

^a^ Each encounter included consent, questionnaire and nasal swab and reflects a unique episode, not a unique participant. In the first year of the study, there were 649 unique individuals and 825 encounters, while in the second and third years there were 14,464 encounters from 3281 unique participants.

^b^ RSV, respiratory syncytial virus.

^c^ Comorbidites were self‐reported; participants could have more than 1 comorbidity.

Nasal specimens collected from 35 encounters tested positive for RSV (15 RSV‐A and 21 RSV‐B), with one participant testing positive for both RSV A and B during one encounter. Nasal specimens from 77 encounters tested positive for influenza (34 influenza A and 44 influenza B), with one participant testing positive for influenza A and B during one encounter. Other viruses were also detected in a total of 9 specimens (25.7%) with RSV and 16 specimens (20.8%) with influenza (Table 2).

**TABLE 2:** Characteristics of viral co‐detections among residents and staff of homeless shelters.

Pathogens	Encounters with at least 1 virus detected (*N* = 1736)	Co‐detection with RSV^a^ (*N* = 9)	Co‐detection with influenza (*N* = 16)
Adenovirus	168	1	2
Bocavirus	14	0	2
Enterovirus	90	1	0
Influenza	82	3	—
A	34	1	—
B	44	2	—
C	5	0	—
Metapneumovirus	29	1	0
Parainfluenza (1–4)	44	0	0
Rhinovirus	1102	3	3
RSV	35	—	3
Seasonal coronavirus	156	4	6
HCoV‐229E	37	0	0
HCoV‐NL63	101	2	4
HCoV‐HKU1	11	0	0
HCoV‐OC43	2	0	0
229E or OC43	37	2	2
SARS‐CoV‐2	133	0	1

^a^ RSV, respiratory syncytial virus.

The most common symptoms in both participants with RSV and influenza infections were cough (89% and 71%, respectively) and rhinorrhea (86% and 69%, respectively) (Table 3), followed by myalgias (54%), sore throat (51%), and fatigue (49%) for RSV, and fevers (47%), fatigue (40%) and myalgias (35%) for influenza. Four RSV infections (11%) were asymptomatic at the time of enrollment, as were 11 (14%) of influenza virus infections.

**TABLE 3:** Symptoms reported by participants with encounters for which RSV or influenza was detected.

Symptoms	All encounters (*n* = 15,364)	Encounters with RSV detected (*n* = 35)	Encounters with influenza detected (*n* = 77)
	*n* (%)	*n* (%)	*n* (%)
None	11 709 (76.2)	4 (11.4)	11 (14.3)
Rhinorrhea	2514 (16.4)	30 (85.7)	53 (68.8)
Cough	2169 (14.1)	31 (88.6)	55 (71.4)
Sore throat	1320 (8.6)	18 (51.4)	22 (28.6)
Fatigue	1552 (10.1)	17 (48.6)	31 (40.3)
Myalgias	1435 (9.3)	19 (54.3)	27 (35.1)
Headaches	1342 (8.7)	12 (34.3)	25 (32.5)
Shortness of breath	820 (5.3)	15 (42.9)	16 (20.8)
Nausea or vomiting	904 (5.9)	6 (17.1)	25 (32.5)
Subjective fevers	1045 (6.8)	13 (37.1)	36 (46.8)
Sweats	548 (3.6)	3 (8.6)	15 (19.5)
Chills	576 (3.8)	3 (8.6)	17 (22.1)
Diarrhea	646 (4.2)	8 (22.9)	18 (23.4)
Ear pain or discharge	319 (2.1)	2 (5.7)	4 (5.2)
Rash	261 (1.7)	2 (5.7)	4 (5.2)

A substantial proportion of encounters with symptomatic RSV (39%) and influenza (58%) reported that their illness significantly impacted their ability to perform their regular activities. Three (10%) of symptomatic participants (all adults) with RSV and six (9%) of symptomatic individuals with influenza reported having seen a doctor in the previous week for their symptoms; an additional 10% with RSV (*n* = 3, including two children) sought care in an emergency department.

Specimens positive for RSV had OpenArray Ct values ranging between 13.0 and 27.9 (median 23.1, *n* = 35 specimens). Near‐complete RSV genomes with <10% Ns (ambiguous bases called in regions with less than 5× coverage) were obtained for seven specimens including one RSV‐A and 6 RSV‐B specimens from three shelters (Table S1). The sample size was too small to observe clustering by shelter. The RSV‐A‐positive specimen fell in the lineage A.23, and all RSV‐B fell in the B.6 lineage.

Two encounters with RSV (6%) and one encounter with influenza (1.3%) were among shelter staff. During a 30‐day period in January and February 2019, we documented eight encounters with swabs testing positive for RSV‐B and one testing positive for RSV‐A in a single adult men's shelter. Individual RSV‐B infections were detected 0 to 8 days apart.

**FIGURE 1:**
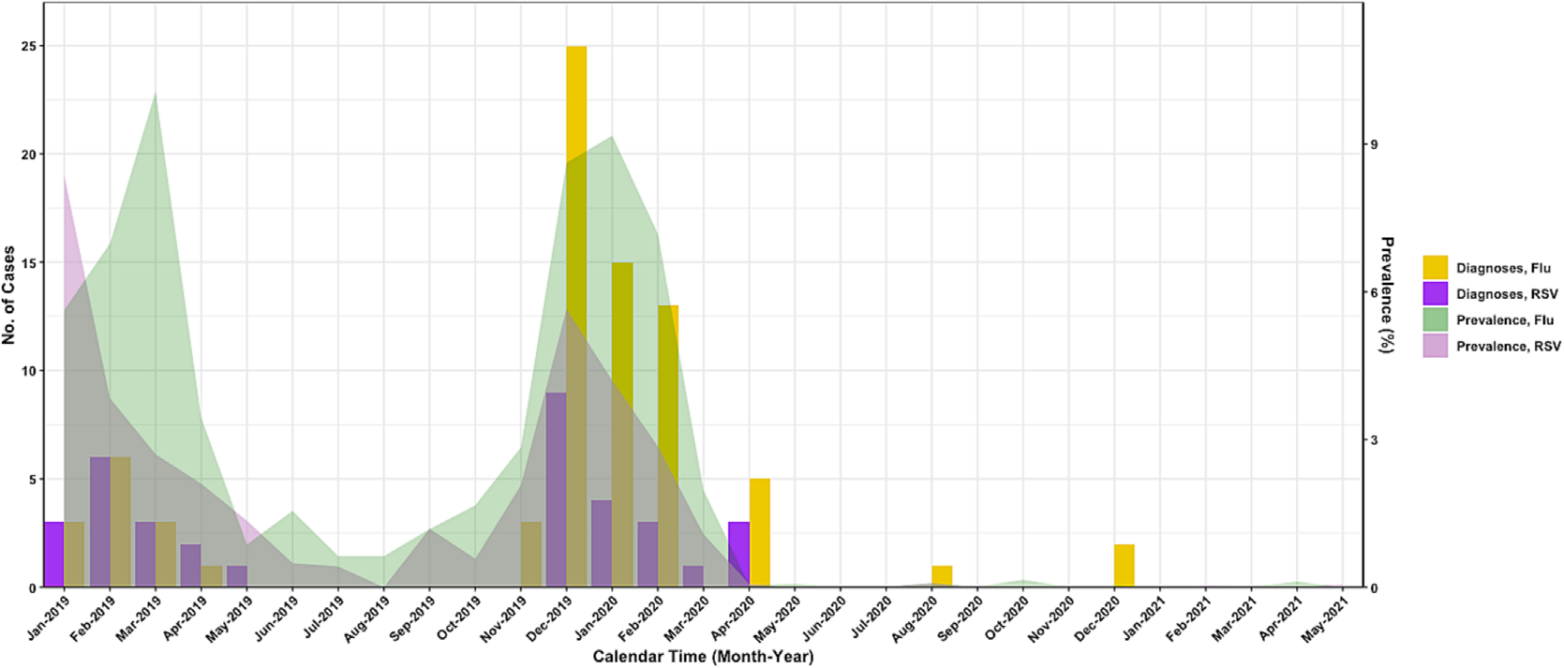
Confirmed respiratory syncytial virus and influenza detections in shelters in Seattle‐King County, WA 2019–2021 compared with overall prevalence of RSV and influenza in the Seattle Flu Study during the same time. Yellow and purple bars show the number of influenza and respiratory syncytial virus detections in shelters each month. The light green and purple shaded areas show the prevalence of influenza and respiratory syncytial virus detections in the Seattle Flu Study overall during the same time period. Prevalence was calculated as the percent of all Seattle Flu Study specimens with RSV or influenza detected, aggregated by week across Seattle Flu Study collection strategies.

RSV‐positive encounters peaked in February 2019 and again the following season in December 2019, with no detections after April 2020, similar to community epidemic curves (Figure 1).

## 4 DISCUSSION

In this study of residents and staff of urban homeless shelters, RSV and influenza presented with comparable symptoms and disease burden, with overlapping clinical presentations and seasonality, and similar levels of medical care‐seeking.

Limited data describing RSV infections among PEH suggests that homelessness is a risk factor for hospitalization with RSV infection.^14^ If targeted treatments for RSV become available, providing access to testing for RSV will be a key step in ensuring timely access to antivirals for patients in this vulnerable group. Our data showed substantial overlap in the seasonal peaks and clinical presentations of RSV and influenza, suggesting that rapid, accurate testing will be necessary because clinical diagnosis is not sufficient to ascertain the cause of acute respiratory disease.

We found respiratory viral co‐detections in 26% of encounters with RSV and 21% with influenza. Data on the frequency of co‐detections in community settings is limited; one household study detected ≥2 viruses in 30 of 237 (13%) specimens.^10^ Our higher detection rate suggests that shelter environments may predispose to the acquisition of multiple concurrent viruses; alternatively, highly symptomatic individuals may have been both more likely to enroll and more likely to be coinfected with multiple viruses. However, the disappearance of RSV in our study following the implementation of COVID‐19 mitigation measures suggests that reducing the burden of respiratory viruses is feasible in this setting with nonpharmaceutical interventions (NPIs) such as routine testing and availability of isolation and quarantine units.^15^

Six percent of RSV‐positive encounters were among shelter staff members, highlighting the role non‐residents of shelters may play in disease transmission within the shelter setting. Future NPIs will need to take into account not only shelter residents, but also shelter workers, who might transmit viruses to vulnerable shelter populations.

In early 2019, eight specimens tested positive for RSV‐B in a short time period in a single shelter. Although sequencing was successful on only four of these eight specimens, this RSV‐B cluster is suggestive of phylogenetic relatedness, supporting the theory that these infections may have involved within‐shelter transmission events.

Limitations of this study include the lack of concurrent data from a stably housed population for comparison, and a lack of follow‐up data on participants, such that we were unable to distinguish between asymptomatic, pre‐symptomatic and post‐symptomatic infections. Also, we lacked longitudinal data; individual participants were counted separately for each encounter, limiting our results to individual encounters rather than unique participants. Furthermore, enrollment was voluntary and so not all residents were sampled.

Strengths include our multi‐pathogen surveillance with associated symptom data, which enabled clinical and virologic characterization of illness in a population in whom there is almost no data to date. Furthermore, our study involved persons of all ages and multiple racial, ethnic, and socioeconomic backgrounds.

RSV infection may soon be preventable or treatable with novel vaccines and therapeutics, and the medical and social vulnerabilities experienced by PEH make them an important potential target group for these future interventions.

## AUTHOR CONTRIBUTIONS

**Denise J McCulloch:** Conceptualization; formal analysis; methodology; project administration; writing—original draft; writing—review and editing. **Julia Rogers:** Conceptualization; data curation; investigation; methodology; project administration; writing—review and editing. **Yongzhe Wang:** Formal analysis; methodology; writing—review and editing. **Eric James Chow:** Conceptualization; investigation; writing—review and editing. **Amy Link:** Project administration; writing—review and editing. **Caitlin Wolf:** Project administration; writing—review and editing. **Timothy M Uyeki:** Supervision; writing—review and editing. **Melissa A Rolfes:** Writing—review and editing. **Emily Mosites:** Writing—review and editing. **Jaydee Sereewit:** Investigation; project administration; validation; writing—review and editing. **Jeffrey S Duchin:** Writing—review and editing. **Nancy Sugg:** Writing—review and editing. **Alexander Greninger:** Investigation; methodology; resources; supervision; validation; writing—review and editing. **Michael Boeckh:** Supervision; writing—review and editing. **Janet Englund:** Methodology; supervision; writing—review and editing. **Jay Shendure:** Methodology; resources; supervision; writing—review and editing. **James P Hughes:** Methodology; supervision; writing—review and editing. **Lea Starita:** Conceptualization; data curation; investigation; methodology; resources; supervision; validation; writing—review and editing. **Pavitra Roychoudhury:** Data curation; formal analysis; investigation; methodology; validation; writing—review and editing. **Helen Chu:** Conceptualization; investigation; methodology; resources; supervision; writing—review and editing.

## CONFLICT OF INTEREST STATEMENT

Dr. E. Chow reported honoraria from Providence Health & Services, Renton, Washington for presentations on COVID‐19. Dr. P. Roychoudhury reported honoraria from The Bill & Melinda Gates Foundation and Association for Molecular Pathology for presentations on COVID‐19. Dr. M. Boeckh reported consulting with GlaxoSmithKline and Janssen and has received research support from Janssen, outside the submitted work. Dr. A. Greninger reports contract testing from Abbott and research support from Gilead and Merck. Dr. J. Englund reported consulting with Sanofi Pasteur, AstraZeneca, and Meissa Vaccines, and has received research funding from AstraZeneca, GlaxoSmithKline, Merck, and Pfizer outside the submitted work. Dr. H. Chu reported consulting with Ellume, Pfizer, The Bill and Melinda Gates Foundation, Glaxo Smith Kline, Abbvie, and Merck. She has received research funding from Gates Ventures, Sanofi Pasteur, and support and reagents from Ellume and Cepheid outside of the submitted work. All other authors report no conflicts of interest. The findings and conclusions in this report are those of the authors and do not necessarily represent the official position of the Centers for Disease Control and Prevention.

## ETHICS STATEMENT

This study was approved by the University of Washington Institutional Review Board. Participants or their proxies (if aged <18) completed informed consent prior to study participation.

### PEER REVIEW

The peer review history for this article is available at https://www.webofscience.com/api/gateway/wos/peer-review/10.1111/irv.13166.

## Supporting information


**Table S1.** Genbank accession numbers for respiratory syncytial virus (RSV) sequences.

## Data Availability

The data that support the findings of this study are available from the corresponding author upon reasonable request.
