# Supplementary material for: Respiratory syncytial virus and other respiratory virus infections in residents of homeless shelters – King County, Washington, 2019–2021
Source: Influenza Other Respir Viruses. 2023 Jun 19;17(6):e13166. doi: 10.1111/irv.13166 (PMC10279995; doi:10.1111/irv.13166)
Supplement: Supplementary file 1 — Table S1. Genbank accession numbers for respiratory syncytial virus (RSV) sequences. [file IRV-17-e13166-s001.docx]

**APPENDIX**

**Supplemental Methods**

Specimens positive for RSV were sequenced using a shotgun metagenomic or hybridization capture approach as described previously.^1,2^ Briefly, RNA was extracted from specimens using the Roche MagnaPure 96 DNA and viral NA small volume kit, Viral NA Universal SV 4.0 protocol (200 μL input, 50 μL elution). RNA was DNase treated using the Turbo DNA-Free kit (Thermo Fisher). First-strand cDNA was synthesized using Superscript IV (Thermo Fisher) and random hexamers (Integrated DNA Technologies), and second-strand synthesis was performed with Sequenase version 2.0 DNA polymerase (Thermo Fisher). The resulting double-stranded cDNA was purified using AMPure XP beads (Beckman Coulter). Libraries were constructed using the Nextera DNA Flex pre-enrichment kit (Illumina) and cleaned using 0.8 volumes of AMPure XP beads. For samples where genomes could not be recovered using shotgun sequencing, we performed hybridization capture using biotinylated oligonucleotide probes (Illumina Respiratory Virus Oligo Panel). The resulting libraries were sequenced on an Illumina Novaseq using a 1x100 read format or Illumina Nextseq 2000 using 2x150 reads.

Consensus genomes were generated using a custom bioinformatic pipeline (<https://github.com/greninger-lab/revica>). Briefly, raw reads are trimmed with Trimmomatic (v0.39) using the settings ILLUMINACLIP:2:30:10:1:true, SLIDINGWINDOW: 4:20, LEADING: 3, TRAILING: 3, MINLEN: 35. Trimmed reads are mapped to a multi-fasta reference containing complete genomes of multiple respiratory viruses using BBMap (v38.96). The reference with the highest median coverage was selected as the initial reference for consensus calling. Trimmed reads were then mapped again to the initial reference using BBMap with a strict max indel of 9. The resulting alignment was used to call a consensus genome using Samtools (v.1.15) and iVar (v1.3.1). A minimum coverage of 3, a minimum base quality of 15, and a minimum frequency threshold of 0.6 were required to call consensus. Regions with less than the minimum coverage were called Ns. This process was iterated for a total of 3 times and leading and trailing Ns were trimmed to generate a final consensus.

**Supplemental references**

1. Chow EJ, Casto AM, Rogers JH, Roychoudhury P, Han PD, Xie H, Mills MG, Nguyen TV, Pfau B, Cox SN, Wolf CR, Hughes JP, Uyeki TM, Rolfes MA, Mosites E, Shim MM, Duchin JS, Sugg N, Starita LA, Englund JA, Chu HY. The clinical and genomic epidemiology of seasonal human coronaviruses in congregate homeless shelter settings: A repeated cross-sectional study. Lancet Reg Health Am. 2022 Nov;15:100348. doi: 10.1016/j.lana.2022.100348. Epub 2022 Aug 18. PMID: 35996440; PMCID: PMC9387177.
2. Chow EJ, Casto AM, Rogers JH, Roychoudhury P, Han PD, Xie H, Mills MG, Nguyen TV, Pfau B, Cox SN, Wolf CR, Hughes JP, Uyeki TM, Rolfes MA, Mosites E, Shim MM, Duchin JS, Sugg N, Starita LA, Englund JA, Chu HY. The clinical and genomic epidemiology of seasonal human coronaviruses in congregate homeless shelter settings: A repeated cross-sectional study. Lancet Reg Health Am. 2022 Nov;15:100348. doi: 10.1016/j.lana.2022.100348. Epub 2022 Aug 18. PMID: 35996440; PMCID: PMC9387177.

**Supplemental Table.** Genbank accession numbers for respiratory syncytial virus (RSV) sequences

| Sequence name | Collection date | Genbank | Lineage |
| --- | --- | --- | --- |
| hRSV/A/USA/51175/2020 | 1/6/2020 | OQ331220 | A.23 |
| hRSV/B/USA/65fce/2019 | 1/29/2019 | OQ331211 | B.6 |
| hRSV/B/USA/14bf4/2019 | 1/31/2019 | OQ331210 | B.6 |
| hRSV/B/USA/d5493/2019 | 2/12/2019 | OQ331219 | B.6 |
| hRSV/B/USA/81cf6/2019 | 2/12/2019 | OQ331212 | B.6 |
| hRSV/B/USA/64329/2019 | 12/12/2019 | OQ331216 | B.6 |
| hRSV/B/USA/d202e/2019 | 12/26/2019 | OQ331218 | B.6 |
